# Lipopolysaccharides from Commensal and Opportunistic Bacteria: Characterization and Response of the Immune System of the Host Sponge *Suberites domuncula*

**DOI:** 10.3390/md13084985

**Published:** 2015-08-07

**Authors:** Johan Gardères, Gilles Bedoux, Vasiliki Koutsouveli, Sterenn Crequer, Florie Desriac, Gaël Le Pennec

**Affiliations:** University of South Brittany, EA 3884, LBCM, IUEM, F-56100 Lorient, France; E-Mails: jgarderes@mnhn.fr (J.G.); gilles.bedoux@univ-ubs.fr (G.B.); vassiakouts88@gmail.com (V.K.); sterenn.crequer@univ-ubs.fr (S.C.); floriedesriac@hotmail.fr (F.D.)

**Keywords:** lipopolysaccharide, symbiosis, *Porifera*, sponge-associated bacteria, immunity, macrophage-expressed gene

## Abstract

Marine sponges harbor a rich bacterioflora with which they maintain close relationships. However, the way these animals make the distinction between bacteria which are consumed to meet their metabolic needs and opportunistic and commensal bacteria which are hosted is not elucidated. Among the elements participating in this discrimination, bacterial cell wall components such as lipopolysaccharides (LPS) could play a role. In the present study, we investigated the LPS chemical structure of two bacteria associated with the sponge *Suberites domuncula*: a commensal *Endozoicomonas* sp. and an opportunistic *Pseudoalteromonas* sp. Electrophoretic patterns indicated different LPS structures for these bacteria. The immunomodulatory lipid A was isolated after mild acetic acid hydrolysis. The electrospray ionization ion-trap mass spectra revealed monophosphorylated molecules corresponding to tetra- and pentaacylated structures with common structural features between the two strains. Despite peculiar structural characteristics, none of these two LPS influenced the expression of the macrophage-expressed gene *S. domuncula* unlike the *Escherichia coli* ones. Further research will have to include a larger number of genes to understand how this animal can distinguish between LPS with resembling structures and discriminate between bacteria associated with it.

## 1. Introduction

Sponges are holobionts as defined by Zilbner-Rosenberg and Rosenberg [1]. Indeed, they host microorganisms transiently and permanently, including bacteria, archaea, algae and fungi [2,3] with which they maintain permanent interactions [4,5,6,7]. Bacteria can be a source of feeding [8,9,10], pathogenicity [11,12,13] or symbiotism [14,15,16]. Accordingly, sponges have to discriminate, to tolerate and to defend themselves against prokaryotes [17,18]. To achieve these aims, these invertebrates have an appropriate and efficient innate immune system similar to that of higher vertebrates. This immune system recognizes and efficiently fights against pathogens by carrying out a dedicated defense pathway [19,20,21,22,23]. 

About 47 phyla of bacteria inhabit the sponges [24]. Some of these bacteria are sponge-specific and are therefore grouped in a phylum named *Poribacteria* [24,25,26]. Sponge-associated bacteria can be acquired from the environment but can also be vertically transmitted via the gametes [27]. Nevertheless, a recognition mechanism must exist in sponges to discriminate between the bacterial partners of the symbiosis *sensu* de Bary [28], to induce tolerance to specific bacteria and to avoid over proliferation of others. The communication pathways between the host and the prokaryotes remain to be elucidated. Gardères *et al.* demonstrated that sponge-associated bacteria are able to produce bacterial communication molecules, the *N*-Acyl homoserine lactones (AHLs), within the sponge *Suberites domuncula* [29]. These molecules act on sponge cells by inhibiting the immune and apoptotic systems and thus by limiting the immune response [7]. At the same time, the sponge perceives these molecules and increases the *de novo* production of proteins involved in endocytosis in order to likely allow the control of the resident bacterial population [7]. Molecules constituting the bacterial cell wall such as the pathogen-associated molecular patterns (PAMPs) found in the outer membrane of Gram-negative and Gram-positive bacteria, may have an important function in the recognition of prokaryotes by the sponge. Besides, some membrane proteins of *Poribacteria* possess eukaryotic protein domains involved in protein–protein interactions in eukaryote extracellular matrix and cell adhesion [30,31], potentially playing a role in this recognition mechanism.

Lipopolysaccharides (LPS) is a PAMP and constitute one of the main components of the Gram-negative bacterial outer membrane. They are recognized by Toll-Like Receptors (TLRs) present in Metazoan in particular *Porifera* [23]. TLRs play a key role in the activation of the innate immune system in the presence of PAMPs [32,33,34]. As a result, the pro-apoptotic caspases and the anti-apoptotic regulators of the Bcl-2 family participate in defense mechanisms against bacteria [35,36]. LPS consist of three domains: a membrane-associated lipid A, a core oligosaccharide, and an *O*-antigen moiety. They are usually vectors of inflammation in vertebrates [37]. Furthermore, they play a significant role in symbiont settlement, such as in the association between the bobtail squid *Euprymna scolopes* and the bacterium *Vibrio fischeri*. The presentation of LPS of this bacterium in the crypts of the light organ triggers an apoptotic process, leading to controlled morphological changes of this organ of the juvenile squid [38]. In the human gut, the commensal bacterioflora helps to educate the local immune defense system by presenting immunogenic molecules, including LPS, to intestinal epithelial cells and thus activate the defense against pathogen bacteria [39].

The demosponge *S. domuncula* dedicates a specific receptor, the *S. domuncula* LPS Interacting Protein (SLIP) for the recognition of LPS [21]. The fixation of *Escherichia coli* LPS to the SLIP promotes its dimerization and then the recruitment of an adaptive protein, MyD88 [21]. This sponge LPS-related pathway involves the activation by phosphorylation of two mitogen-activating protein kinases, p38 and Jun *N*-terminal Kinase [40]. A set of effective molecules are up-regulated, including the oligoadenylate synthase involved in RNA degradation [41], a perforine-like similar to the macrophage-expressed gene (MPEG) efficient against Gram-negative bacteria [21], a lectin active against *Staphylococcus aureus* and *E. coli* [42], and the alkyl-dihydroxyacetonephosphate synthase responsible for the synthesis of two antimicrobial compounds, the lyso-platelet-activating factors (lyso-PAF)1-*O*-hexadecyl-*sn*-glycero-3-phosphocholine and the 1-*O*-octadecyl-*sn*-glycero-3-phosphocholine [43]. Similar molecules involved in the LPS-signaling pathway were also found in the genome of the demosponge *Amphimedon queenslandica* [44].

In the present study, we investigated how the sponge host immune system behaves when challenged by purified LPS isolated from two *Suberites domuncula*-associated bacteria.

## 2. Results

### 2.1. Identification of Sponge-Associated Bacteria

**Figure 1:**
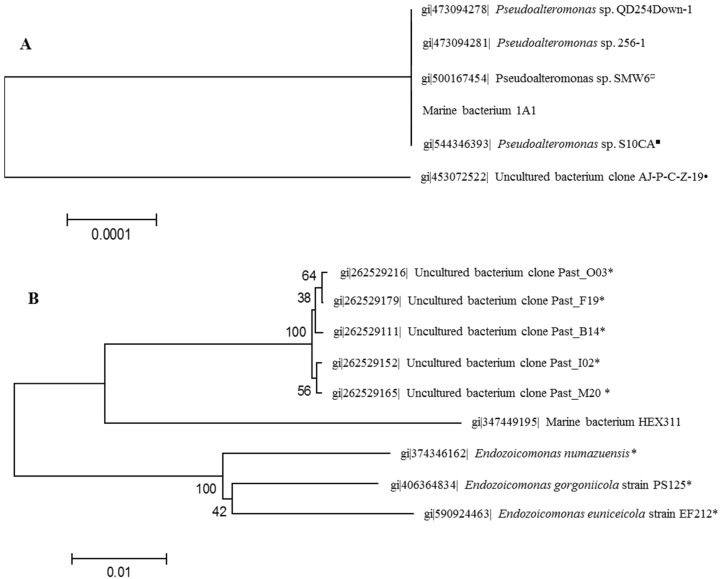
Neighbor-joining phylogenetic trees generated by analyzing partial 16S rDNA gene sequences of *Suberites domuncula*-associated bacteria *Pseudoalteromonas* sp. 1A1 (**A**) and *Endozoicomonas* sp. HEX311 (**B**). Each entry is preceded by its GenBank accession number. The trees were constructed using maximum composite likelihood and pairwise deletion. Percentage bootstrap values (50% only) from 1000 re-samplings are indicated at each node. Bar, 2% estimated sequence divergence. The symbols following the name of bacteria indicate the association of these bacteria with eukaryotic organisms: *asterisk* corals, *filled circle* echinoderms, *filled square* poriferans, *open circle* fishes.

The bacteria were isolated from a healthy and an unhealthy specimen of *S. domuncula*, in a liquid incubation Zobell medium, which was then spread onto corresponding agar media, *i.e.*, with or without sponge extract. Firstly, the most common morphotype among the colonies of released bacteria from the unhealthy specimen was chosen as the opportunistic bacterium and named 1A1. Infectivity of this bacterium was controlled by a re-contamination of sponge (Figure S1). Within 16 h, the culture medium became whitish due to the bacterial proliferation and, after 36 h a white biofilm had developed on the sponge pinacoderm; after 48 h, brown spots were present on sponge cell surface. The result of the sequencing of the 16S rDNA gene indicated that it belongs to the group of *Pseudoalteromonas* (Figure 1A). Secondly, a colony growing only on sponge extract-supplemented Zobell agar medium and isolated from a healthy sponge was selected as the commensally bacterium and named HEX311. Infection experiments with this bacterium did not impact the sponge morphology (Figure S1). The sequencing of the 16S rDNA gene of this bacterium identified it as belonging to the genus *Endozoicomonas* (Figure 1B). However, this bacterium is phylogenetically more related to uncultivable coral-associated bacteria identified by metagenomic approaches [45], rather than to the *Endozoicomonas* genus (Figure 1B).

### 2.2. Electrophoretic Pattern of Bacterial LPS

**Figure 2:**
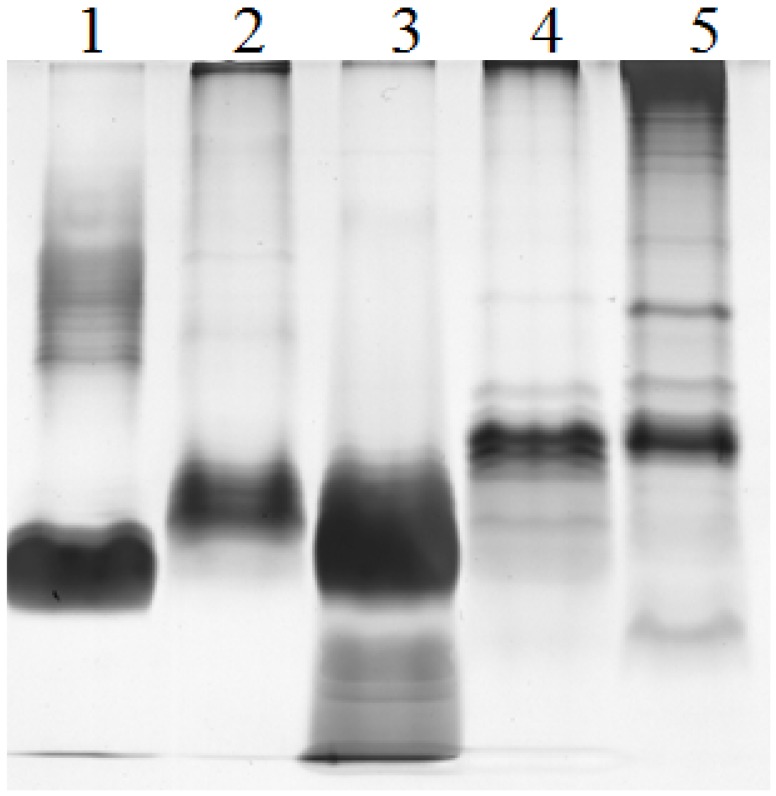
Silver-stained SDS-polyacrylamide gels showing the LPS profiles of strains *Pseudomonas aeruginosa* PAO1 (PAO1 wild-type, lane 1), *Salmonella enterica* serovar Enteritidis (SE CIP, lane 2), *Escherichia coli* 0111:B4 (lane 3), *Endozoicomonas* sp. HEX311 (lane 4) and *Pseudoalteromonas* sp. 1A1 (lane 5).

LPS isolated from *E. coli, Pseudoalteromonas* sp. 1A1 and *Endozoicomonas* sp. HEX311 strains were separated by SDS-PAGE. LPS from *Pseudomonas aeruginosa* PAO1 (wild type) and *Salmonella enterica* serovar Enteritidis (SE CIP) were used as references. The LPS of each strain were characterized by a SDS-PAGE profile, which differed from the four others (Figure 2). PAO1 (lane 1) and *Pseudoalteromonas* sp. 1A1 (lane 5) profiles appeared as a smooth-type LPS with *O*-antigen bands (half top of the gel) and a lipid A-core band (half bottom of the gel), whereas SE CIP (lane 2), *E. coli* (lane 3) and *Endozoicomonas* sp. HEX311 (lane 4) were not silver stained in top part of the gel. LPS of *Endozoicomonas* sp. HEX311 presented bands corresponding to a truncated LPS structure devoid of a long *O*-chain.

### 2.3. Carbohydrate Composition

The neutral carbohydrate composition was studied on *P. aeruginosa* PAO1, *S. enterica* Serovar Enteritidis, *Pseudoalteromonas* sp. 1A1 and *Endozoicomonas* sp. HEX311 LPS. Other sugars (amino sugars, uronic acids) were not identified here. Glucose (Glc), rhamnose (Rha), galactose (Gal), mannose (Man) and arabinose (Ara) residues were determined by high-performance anion-exchange chromatography (HPAEC) among the four strains. Glc was identified in PAO1, SE CIP and *Pseudoalteromonas* sp. 1A1, Rha in PAO1, SE CIP and *Endozoicomonas* sp. HEX311. Man was only present in SE CIP, whereas LPS from *Endozoicomonas* sp. HEX311 contained Rha, Gal and Ara. 

### 2.4. ESI-MS/MS of Lipid A

**Figure 3:**
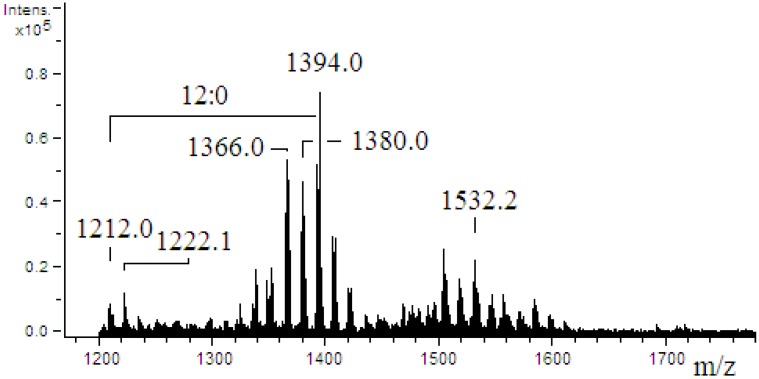
ESI-mass spectrum obtained in the negative ion mode of lipid A from *Pseudoalteromonas* sp. 1A1. No additional peak was detected under *m*/*z* 1200. The mass difference (182 amu) between the *m*/*z* 1394.0 and 1212.0 corresponds to a 12:0 acyl chain.

The mass spectrum of *Pseudoalteromonas* sp. 1A1 lipid A (Figure 3) revealed 15 singly charged ion peaks [M − H]^−^ distributed through three clusters with one major peak within each cluster (*m*/*z* 1222.1, 1394.0 and 1532.2). Accordingly, a molecular mass of 1394.8 Da was calculated for the formula C_72_H_134_O_24_N_2_P, using the isotopic average mass for each atom. 

**Figure 4:**
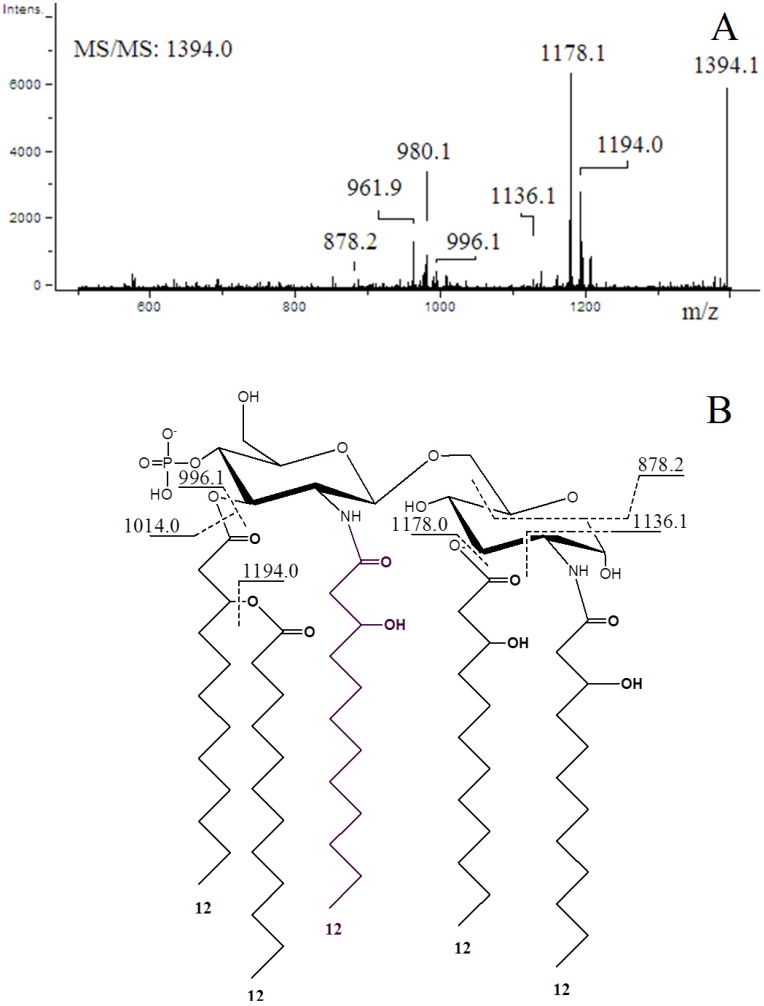
Negative ion ESI-MS/MS spectrum of the [M − H]^−^ precursor ion *m*/*z* 1394.0 of a monophosphorylated lipid A from *Pseudoalteromonas* sp. 1A1. The structure of the precursor ion and fragmentation pattern with expected *m*/*z* values are shown.

The ion-trap MS^2^ analysis was performed on the most abundant peak (*m*/*z* 1394.0) to check its structure (Figure 4). The tandem mass spectrum (Figure 4A) showed peaks at *m*/*z* 1194.0 and 1178.1 as a consequence of the respective losses of 12:0 (200.1 amu) and 12:0(3-OH) (216.2 amu). The structures and the fragmentation pattern of the lipid A with expected *m*/*z* values are shown in Figure 4B. The *m*/*z* 1394.0 ion gave a ^0,2^A_2_ fragment ion at *m*/*z* 1136.1, according to the nomenclature by Costello and Vath [46]. Thus, the nature of the acyl chain located on the C-2 position of the reducing glucosamine residue was determined by the rest of the mass. This backbone was substituted at the C-2 position by *N*-linked 12:0(3-OH). This peak at *m*/*z* 1136.1 clearly indicated that the phosphate group was located on the non-reducing glucosamine at the C-4′ position, and that the acyl chains 12:0(3-OH) and 12:0 were linked at the C-3, C-2′ or C-3′ positions. The spectrum also contained peaks at *m*/*z* 878.2 ion corresponding to a ^0,4^A_2_ fragment ion confirming the linkage of a 12:0(3-OH) at the C-3 position on the reducing glucosamine.

**Figure 5:**
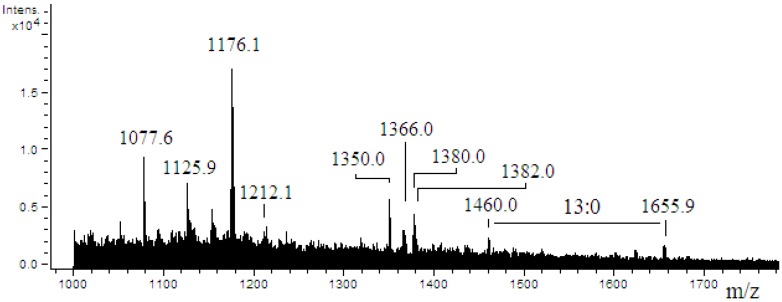
ESI-mass spectrum obtained in the negative ion mode of lipid A from *Endozoicomonas* sp. HEX311.

The mass spectrum of *Endozoicomonas* sp. HEX311 lipid A (Figure 5) revealed 10 singly charged ion peaks [M − H]^−^ with one major peak at *m*/*z* 1176.1. Among the observed peaks, three peaks at *m*/*z* 1212.1, 1366.0 and 1380.0 were detected in addition to minor peaks centered at *m*/*z* 1460.0 and *m*/*z* 1655.9 with a mass difference corresponding to a 13:0 acyl chain (196.0 amu). The low intensity of the signal (total ion current) did not allow the isolation of any parent ion and a MS/MS fragmentation step.

### 2.5. Stimulation of Sponge Primmorphs with LPS 

**Figure 6:**
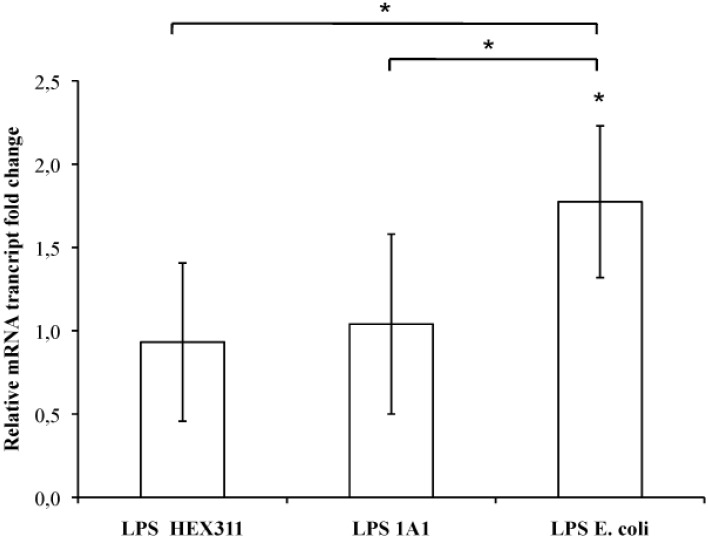
Ratio of the MPEG gene transcripts from *Suberites domuncula* primmorph cultures stimulated/unstimulated with lipopolysaccharides from sponge-associated bacteria *Endozoicomonas* sp. HEX311 and *Pseudoalteromonas* sp. 1A1 and from the bacterium *Escherichia coli*. The mRNA amounts from five stimulated *S. domuncula* primmorphs were evaluated by quantitative reverse transcription-PCR and compared to non-stimulated primmorphs. Values above and below 1 showed a higher and a lower mRNA level in the presence of LPS, respectively. Significant expression changes (*p* < 0.05) in the presence of LPS are pointed out by an asterisk.

Five cultures of primmorph prepared from five specimens of *S. domuncula* were incubated in the presence of purified LPS from *Endozoicomonas* sp. HEX311, *Pseudoalteromonas* sp. 1A1 and *E. coli*. After 16 h of incubation, the expression of the MPEG gene statistically increased by an average ratio of 1.74 when the primmorphs were cultivated in the presence of *E. coli* LPS, whereas it did not change significantly in the presence of LPS from *Endozoicomonas* sp. HEX311 and *Pseudoalteromonas* sp. 1A1 (average ratio of 0.93 and 1.04, respectively) (Figure 6). The expression of the MPEG gene turned out to be dependent on the LPS origin. 

## 3. Discussion

Marine sponges harbor a large bacterioflora including mutual, commensal and opportunistic bacteria while some pathogenic bacteria, rarely mentioned in the literature, compromise the health of these animals [47,48,49,50]. Anyway, the transient or permanent associations with bacteria lead to a complex process of molecular interactions between microorganisms and hosts. Sponges have to discriminate, besides the identification of bacteria with food fate and resident bacteria, between symbiotic, opportunist and pathogenic bacteria and to answer by an appropriate behavior. For example, sponges have to regulate the proliferation of opportunistic bacteria so that they do not become invasive, surpass the sponge immune defense and thus turn pathogenic. The bacterial biomass control could involve an early detection of circulating and/or membrane-borne molecules produced by bacteria. LPS could participate in this general scheme of identification of bacteria by sponges, like AHLs [7] and eukaryotic protein domains do [30,31].

### 3.1. Identification of Sponge-Isolated Bacteria

The commensal bacterium *Endozoicomonas* sp. HEX311 isolated onto sponge extract-supplemented Zobell agar medium requires host molecules to grow. Furthermore, experimental infections of *S. domuncula* with this bacterium did not trigger any morphological change at the sponge surface. This cultivable bacterium could represent a new mutual bacterial species associated with marine invertebrates as mentioned by Sunagawa *et al.* [45]. 

The opportunistic bacterium 1A1, isolated from an unhealthy *S. domuncula* specimen, infected experimentally other healthy sponges. It is phylogenetically related to other marine *Pseudoalteromonas* species and, especially to one found in a carnivorous sponge [51]. Besides, considering the pathogenicity of *P. agarivorans* strain NW4327 toward sponges [47] and the proliferation of *Pseudoalteromonas* sp. 1A1 in the presence of sponges, the latter could be considered as a new opportunistic pathogen of the sponge *S. domuncula*. *Pseudoalteromonas* sp. 1A1 is the first pathogenic bacterium associated with this species of sponge to be listed. 

### 3.2. Characterization of Bacterial LPS

The LPS of *Pseudoalteromonas* sp. 1A1 and *Endozoicomonas* sp. HEX311 were extracted, characterized and compared to those of *E. coli*, *P. aeruginosa* PAO1 and *S. enteritidis* SE CIP. Electrophoretic analysis of LPS from the strain PAO1 showed bands identical to the smooth-type LPS previously proposed [52]. LPS of *Pseudoalteromonas* sp. 1A1 displayed high molecular weight bands corresponding to a complete LPS structure carrying *O-*chain.

Results obtained for the carbohydrate composition are consistent with previous studies concerning PAO1 [53,54], SE CIP and *Pseudoalteromonas* [55,56]. LPS of *Endozoicomonas* sp. HEX311 displayed an original carbohydrate composition with Rha, Gal and Ara, confirming that the LPS of *Pseudoalteromonas* sp. 1A1 and *Endozoicomonas* sp. HEX311 exhibit a different oligosaccharide part linked to the lipid A. 

Mass spectrometry analyses performed on the lipid A of *Pseudoalteromonas* sp. 1A1 and *Endozoicomonas* sp. HEX311 established differences. For *Pseudoalteromonas* sp. 1A1, the most abundant high mass ion corresponded to a monophosphorylated pentaacyl structure, containing four 12:0(3-OH) and one 12:0 chains, linked to the diglucosamine backbone. The pentaacylated components appeared to be the major lipid A component, as demonstrated in previous works [56,57]. The fragmentation of the most abundant high mass ion performed showed that both ^0,2^A_2_ and ^0,4^A_2_ cross-ring fragmentations during MS/MS stage, as previously described [57,58]. Two fragmentation mechanisms for the elimination of an acyl chain or acyl-oxyacyl chain from the lipid A backbone have been described by Kussak and Weintraub and Bedoux *et al.* [58,59]. The cyclic charge driven process eliminated the acyl or the acyl-oxyacyl group located at the C-3′ position of the lipid A which possesses a 4′-phosphate group. Then, the elimination of 12:0(3-*O*(12:0)) during MS/MS of the *m*/*z* 1394.1 ion and the lack of 12:0 from the ^0,2^A_2_ and the ^0,4^A_2_ cross-ring fragments permitted the location of a 12:0 chain on the non-reducing glucosamine as previously reported [57]. The mass spectrum of *Endozoicomonas* sp. HEX311 lipid A was characterized by a major peak corresponding to the mass of a tetraacylated lipid A built up of two glucosamine units, one phosphate, three 12:0 chains and one 14:0 chain. The hydrolysis of LPS by using acetic acid has been shown to cleave the linkage between lipid A and core part and to release monophosphorylated lipid A with the phosphate group located on the non-reducing glucosamine at the C-4′ position [59]. The ESI mass spectrum revealed the sample heterogeneity, which is typical for the constitutive structures of a given lipid A preparation. The common structural features between the lipid A of *Pseudoalteromonas* sp. 1A1 and *Endozoicomonas* sp. HEX311 were a preliminary characterization. Further analysis for the determination of acyl chains distribution of *Endozoicomonas* sp. HEX311 has to be performed. This is the first study carried out on the lipid A structure of *Endozoicomonas* sp. HEX311. 

### 3.3. Effect of LPS on the Expression of the MPEG Gene: Structure–Function Relationship

*In vitro* cultures were used to explore the immune response of the sponge *S. domuncula* in the presence of several LPS. Primmorphs being axenic, it is easy to re-contaminate them with a single bacterium to evaluate its effects on sponge cells. This avoids a cross-reaction between bacteria and erroneous interpretations of the answer of the host due to the presence of numerous prokaryotic species. The expression of the MPEG gene was evaluated in each condition, *i.e.*, in the presence of LPS from *E. coli*, *Pseudoalteromonas* sp. 1A1 and *Endozoicomonas* sp. HEX311. As previously described [21], LPS from *E. coli* stimulated the expression of the MPEG gene after 16 h of incubation whereas the stimulation of culture with LPS from *Endozoicomonas* sp. HEX311 and *Pseudoalteromonas* sp. 1A1 did not modify its expression. If this result can be considered as normal in the presence of LPS of *Endozoicomonas* sp. HEX311 because this bacterium lives at least transiently in the sponge, it is more amazing in the presence of LPS of *Pseudoalteromonas* sp. 1A1. LPS from the opportunistic bacterium *Pseudoalteromonas* sp. 1A1 did not trigger the same response as the one observed with *E. coli* LPS, while this bacterium had the ability to become in certain conditions a pathogen for the sponge. In fact, the regulation of this gene is LPS-dependent and its stimulation can change according to the chemical structure of the LPS [60]. 

#### 3.3.1. The Degree of Acylation of the Lipid A

The lipid A is one of the major components of the LPS, responsible for its inflammatory activity. Besides, the lipid chains, the degree of acylation influence the host immunomodulatory activity [61]. The *Pseudoalteromonas* sp. 1A1 lipid A profile presented a main form of monophosphorylated pentaacyled structure as already listed for different species belonging to the genus *Pseudoalteromonas* [55,56,57,62,63]. The lipid A of *Endozoicomonas* sp. HEX311 was mainly characterized by a tetraacylated form. Besides the major tetraacylated HEX311 lipid A structure, the form centered at *m*/*z* 1366.0 indicated the presence of pentaacylated derivatives. The relatively low acylation of the lipid A of *Pseudoalteromonas* sp. 1A1 and *Endozoicomonas* sp. HEX311 could possibly explain the similar MPEG gene regulation in the presence of the two LPS. Marine bacteria usually displayed a weaker proinflammatory LPS, characterized by shorter acyl chains, less fatty acids and often carrying only a single phosphate group [64]. Moreover, highly acylated lipid A (hexaacylated), including the *E. coli* lipid A, trigger a high inflammation, compared to less acylated lipid A-containing bacteria. Indeed, the tetraacylated lipid A from *Yersinia pestis,* cultivated at 37 °C, engenders a weaker immunoinflammatory response in human macrophages cells than its hexaacylated lipid A-containing LPS, produced when cultivated at 27 °C. This shorter lipid A LPS form does not trigger the TLR4-related pathway and allows the bacteria to escape the immune defense of the host [65]. In the present study, both studied bacteria contained LPS with a lipid A, the degree of acylation of which is less immunostimulatory than these of *E. coli.*

#### 3.3.2. The Position of the Phosphate on the Lipid A

The phosphate position on the lipid A may also contribute to the level of inflammation [61,66]. Indeed, both *Bacteroides thetaiotaomicron* and *Porphyromonas gingivalis* are phylogenetically related pathogens involved in the chronic oral inflammatory disease periodontitis. The TLR4-stimulatory lipid A of *B. thetaiotaomicron* bears a 1-phosphate, while the TLR4-evasive lipid A from *P. gingivalis* LPS bears a 4′-phosphate. This single difference changes the potencies of LPS to stimulate the TLR4-related pathway activation [66]. Besides, LPS with a non-phosphorylated lipid A does not produce any immunostimulatory activity via TLR pathways, as illustrated by the *Eurypmna* *scolopes*/*Vibrio fischeri* association [67]. The lipid A borne by the LPS of *V. fischeri*, which already exhibits an uncommon phosphoglycerol moiety [68] and an unusual carbohydrate composition [69], is dephosphorylated by an alkaline phosphatase produced by the host [70]. This process avoids the symbiont to trigger a host immune response. Furthermore, LPS of *V. fischeri*, together with the peptidoglycan tracheal cytotoxin, have a particular role in the morphogenesis and immunity regulation in the light organ of the squid [38,71]. LPS of *Endozoicomonas* sp. HEX311 and *Pseudoalteromonas* sp. 1A1 also bear a phosphate on C4′-position on the lipid A. This could explain, at least partially, why both the tested bacteria escape the sponge TLR-related pathway.

#### 3.3.3. The Carbohydrate Composition

The analyses of LPS carbohydrate composition from all the tested strains, *i.e.*, *E. coli*, *Pseudoalteromonas* sp. 1A1 and *Endozoicomonas* sp. HEX311, demonstrated that the carbohydrate composition did not play any crucial role in the immune stimulation. This is confirmed by a previous study pointing out that bacteria from the genus *Pseudoalteromonas*, displaying different carbohydrate structures, presented a similar immunostimulatory behavior [72]. 

## 4. Experimental Section 

### 4.1. Sample Collection

Sponges belonging to the species *Suberites domuncula* were collected by scuba diving at 0–10 m depth during two harvests in Mars 2009 and January 2012 in Roscoff (48°42′48.17′′ N, 3°54′04.28′′ W), Britany, France. Sponges were immediately transferred to the Université de Bretagne-Sud, France, in aquariums under controlled conditions of temperature (12 °C) and luminosity (10–14 h light/dark) in a closed circulating system of natural seawater. Sponges were fed with a protein solution twice a week.

### 4.2. Isolation of Sponge-Associated Bacteria and Culture of Reference Bacteria

Three healthy sponges and one presenting grey spots on its surface present in an aquarium at the time of a bacterial bloom, were used to isolate commensal and opportunistic bacteria, respectively. Sponges were individually cut into small pieces (1 cm^3^) under sterile conditions. Pieces were washed three times with sterile seawater to remove epibiotic organisms. After incubation for 1 min of pieces from unhealthy specimens in 1 mL of sterile natural seawater, the suspension was spread onto Zobell agar medium (1.30 g L^−1^ yeast extract, 6.61 g L^−1^ peptone, 15.00 g L^−1^ agar in sea water, pH 7.4) to isolate opportunist bacteria. After 24 h of culture at 20 °C, the most occurring colony morphotype was picked and streaked onto fresh Zobell medium. The commensal bacteria selected for this study was chosen among *S. domuncula*-isolated bacteria during a previous study, growing on sponge extract-supplemented (10 g L^−1^) Marine broth agar (Difco, Bordeaux, France). 

*Pseudomonas aeruginosa* PAO1 (PAO1), *Salmonella enterica* serovar Enteritidis CIP8297 (SE CIP) strains were grown at 37 °C in Luria Bertani liquid medium (LB) consisting of 1.0% (w:v) tryptone, 1.0% (w:v) NaCl, and 0.5% (w:v) yeast extract, adjusted to pH 7.0 under permanent agitation (140 rpm) during 12–16 h. For standardization of the LPS extraction procedure, the absorbance values at 600 nm of the bacterial suspensions were adjusted to 0.8. 

### 4.3. Identification of Sponge-Associated Bacteria

The 16S rDNA genes were amplified by PCR. Ten microliters of pure bacterial cultures were centrifuged at 12,000 g for 10 min. Supernatants were discarded and bacteria cell pellets were microwaved three times for 10 s, at 900 W, to disrupt cell membranes. Resulting material was suspended into 50 µL of PCR grade water. The prepared total DNA was kept at −20 °C prior to 16S rDNA amplification. 16S rDNA genes were amplified using the universal bacterial primers 8F and 1492R. The reaction mixture (25.0 μL) contained 10.0 μL of Failsafe TM PCR 2X premix D (Epicentre Biotechnologies, Madison, WI, USA), 1.0 μL of each primer (5 μM), 1.0 μL of the bacterial extract, 0.1 μL of MasterAmp Taq DNA polymerase (Epicentre Biotechnologies, Madison, WI, USA) and 11.9 µL of PCR grade water. An initial denaturation step (95 °C for 5 min) was followed by 45 cycles of 95 °C for 30 s, 57.8 °C for 45 s, and 72.0 °C for 90.0 s. A final elongation step was performed at 72.0 °C for 4 min. PCR products were purified and sequenced by Beckman Coulter Genomics (Takeley, UK). The bacterial genus was determined by comparing the amplified sequences of 16S rDNA genes with those listed in the GenBank database using the BLAST software (http://blast.ncbi.nlm.nih.gov). Neighbor-joining phylogenetic trees were constructed using MEGA 6.06 [73] with the 16S rDNA gene sequence of each studied bacterium and the five highest related 16S rDNA sequences found in the GenBank database. Bootstrap tests were performed 1000 times.

### 4.4. Lipopolysaccharide Preparation

*Escherichia coli* 0111:B4 lipopolysaccharides were purchased from Sigma-Aldrich (St. Quentin Fallavier, France). Lipopolysaccharide preparations from strains PAO1, SE CIP, HEX311 and 1A1 were performed according to [74]. Briefly, 100 mL of a 3 day-old-culture of the selected bacteria were centrifuged at 3000 *g* for 10 min at room temperature (RT, namely 20 ± 1 °C). The resulting bacterial pellet was suspended in distillated water (17 mL per g of bacteria) and heated at 65 °C. An equal volume of phenol 90% (v:v) preheated at 65 °C was added to the bacterial suspension. The solution was vigorously mixed for 1 min and further incubated at 65 °C, for 15 min, under permanent agitation. After incubation into an ice bath for 10 min, the solution was centrifuged at 3000 *g* for 45 min, at 4 °C. The upper phase was stored at 4 °C and the lower phase was extracted once again with an equal volume of distillated water at 65 °C with the same procedure. Both aqueous phase were gathered together and dialyzed against distillated water for 3 days (membrane MWCO 4000–6000 Da) to remove remaining molecules of phenol. The solution was then centrifuged at 3000 *g* for 45 min, at 4 °C. The supernatant was frozen at −80 °C before freeze-dried. Dried extract was suspended in 3% (w:v) of distillated water and then centrifuged at 80,000 *g* for 6 h, at 4 °C. The pellet was suspended in one milliliter of distillated water and then centrifuged once again at 105,000 *g* for 3 h, at 4 °C. The final pellet is then suspended in distillated water at a final concentration of 2 mg mL^−1^.

### 4.5. SDS-PAGE and Silver Nitrate Coloration

SDS-PAGE was performed on a 15% (v:v) polyacrylamide gel (acrylamide:bisacrylamide, 37:1) and sodium dodecyl sulfate (SDS) 0.1% (v:v) in a denaturing sample buffer (Tris-HCl buffer 62.5 mM pH 6.8, 2% SDS (w:v), 2 × 10^−3^% (w:v) bromophenol blue, 5% (v:v) mercaptoethanol). Preparative electrophoresis was performed using the Protean III electrophoresis cell (Bio-Rad Laboratories, Hercules, CA, USA). Lipopolysaccharide solution (2 μg of LPS) was loaded onto a polyacrylamide gel column and separated using an electrophoresis buffer (25 mM Tris, 192 mM glycine, SDS 0.1%) with a constant voltage of 80 V for 2 h. A molecular weight standard solution, from 8 to 220 kDa, (Bio-Rad Laboratories, Hercules, CA, USA) was used as a molecular mass calibrator. A solution of *E. coli* polysaccharides (2 μg of LPS) (Sigma-Aldrich, St. Quentin Fallavier, France) was loaded as a control. Gels were washed for 20 s in distillated water and were then incubated into a fixative solution of 40% methanol and 5% acetic acid solution (v:v) for 1 h. The gel was washed twice 20 min in 50% (v:v) ethanol and then twice 5 min in distillated water. Molecules were sensibilized one minute in 0.02% Na_2_S_2_O_4_ (w:v) prior to wash the gel in distillated water. The gel was colored using a 0.1% silver nitrate solution for 20 min and washed twice for 20 s in distillated water. Molecules were then revealed using a developing solution (0.0375% (v:v) formalin, 0.0004% (w:v) Na_2_S_2_O_4_, 6% (w:v) Na_2_CO_3_). Once the appropriated coloration appeared, the gel was washed with a 1% (w:v) glycine solution. The reaction was stopped by incubating the gel for 10 min into a stop solution (1% glycine (w:v), 12% acetic acid (v:v)). The gel was finally washed 10 min in distillated water prior to digitalization (Molecular Imager G800 Calibrated Densitometer, Bio-Rad Laboratories, Marnes-la-Coquette, France) at 400 dpi by Quantity One software (Bio-Rad Laboratories, Marnes-la-Coquette, France).

### 4.6. Carbohydrate Composition

The carbohydrates composition of LPS was determined by High Pressure Anion Exchange Chromatography (Dionex, Courtaboeuf, France). Prior to the analyses, 1.0 ml (2 mg mL^−1^) of samples was hydrolysed for 48 h at 100 °C with 110 µl of 1 M hydrochloric acid in a sealed glass tube. Samples were neutralized with 1 M of sodium hydroxide. The analyses were performed on an analytical column (CarboPac PA1, 4 mm × 250 mm). The elution, 1 mL min^−1^ and 110 bars, was performed in alkaline conditions. Solution A consisted of Milli-Q water, solution B was 0.1 M sodium hydroxide and solution C was 1.0 M sodium acetate/0.1M sodium hydroxide. The elution program corresponded to: 20 min isocratic conditions (solution A/solution B, 80:20), linear gradient (solution C 100%, 0 to 5 min), 5 min isocratic conditions (solution C, 100%) and 20 min isocratic conditions (solution A/solution B, 80:20). Carbohydrates were detected by pulsed amperometry with a detector composed of a silver standard electrode and a gold working electrode. Chromatograms were treated by using Chromeleon^®^ software provided by Dionex (Courtaboeuf, France). Carbohydrates were identified and quantified by using calibration curves of standards (50 µM).

### 4.7. Lipid A Sample Preparation for Mass Spectrometry

Lipid A was prepared by mild acetic acid hydrolysis of crude LPS. LPS were dissolved in a 25 mL round bottomed flask topped with a condenser, at a concentration of 4 mg mL^−1^ in 1% (v:v) acetic acid solution, and heated at 100 °C for 2 h [75]. The precipitated lipid A was recovered by centrifugation (12,000 *g*, 4 °C, 30 min). The pellet was washed twice with distilled water, centrifuged as described above and lyophilized. Lipid A samples were dissolved in a chloroform/methanol (2:1, v:v) solution. Twenty mL of the solution (0.1 mg mL^−1^) were directly introduced into the electrospray ionization interface at a flow-rate of 200 μL h^−1^ by using a syringe pump (Cole-Parmer Instrument Company, Vernon Hills, IL, USA).

### 4.8. Mass Spectrometry

ESI experiments were carried out with an HP-Bruker Esquire-LC ion-trap mass spectrometer (Bruker Daltonik GmbH, Bremen, Germany) equipped with an ESI source, an ion-trap analyzer and Bruker data analysis software. Nitrogen was used as nebulizing gas at a pressure of 30.0 psi. Nitrogen was also used as a heated drying gas at a flow-rate of 7.0 L min^−1^ at 300 °C. In order to assist in the production of droplets and ions, end plate offset voltage was set up to −500 V and the capillary voltage was −4500 V. Calibration of the mass spectrometer was achieved by injection of a tuning mix of phosphine and phosphazine (Bios-Analytique, l’Union, France).

Spectra were recorded in the negative ion mode. The maximum accumulation time was fixed to 0.40 ms and five spectra were accumulated and averaged to display the MS profile. In order to establish the MS^n^ fragmentation pattern, the fragmentation of the precursor ion was initiated with a width of 4 and an amplitude fixed between 1.2 and 1.8 V depending on the total signal intensity.

### 4.9. Sponge Cell Culture and Primmorph Stimulation

Experiments on sponge cell culture were performed in five replicates from five different healthy specimens of *S. domuncula*. For each replicate, sponge cells were isolated for cell culture procedures according to Le Pennec *et al.* [76]. Briefly, sponge pieces (3 cm^2^) were taken from the pinacoderm of specimens and then individually cut into small cubes (1 mm^3^) in Calcium- Magnesium-Free Seawater (CMFSW) (460 mM NaCl, 7 mM Na_2_SO_4_, 10 mM KCl, 10 mM Hepes, 73 mg mL^−1^ of penicillin G potassium, 34.5 mg mL^−1^ of streptomycin sulfate, pH 7.4) containing EDTA (250 mM) under sterile conditions. Fragments of sponges were incubated for 30 min at RT on a rotary shaker to accelerate the dissociation. After collection and filtration of the supernatant containing dissociated cells on a nylon cloth with 20 μm-pores, fresh CMFW-EDTA solution were added to the remaining tissue fragments. They were then incubated for further 30 min and treated in the same conditions as above. Immediately after each 30 min of incubation in CMFSW + EDTA the cell suspension was centrifuged at 800 *g* for 5 min at 20 °C to pellet the cells. The supernatant was discarded and the cell pellet was suspended in CMFSW without EDTA to remove the EDTA. This procedure was repeated once again. The cells from the two incubations were pooled and centrifuged at 800 *g* at RT for 5 min. Finally, the resulting cell pellet was suspended in 0.2 µm-filtered natural seawater collected at the seashore of Ploemeur, Brittany, France. The seawater was supplemented by sea sand (Sigma-Aldrich, St. Quentin Fallavier, France), 10 mM ferrous citrate (Sigma-Aldrich, St. Quentin Fallavier, France), 1 mM of pyruvate (Sigma-Aldrich, St. Quentin Fallavier, France), 73.0 mg mL^−1^ of Penicillin G potassium and 34.5 mg mL^−1^ of Streptomycin sulfate to prevent any bacterial growth. The cell suspension was separated in four fractions of equal volumes which were incubated into Petri dishes, at 15 °C, under permanent gentle shaking. During the first two days of incubation, the cells clumped and aggregated, forming axenic primmorphs. At this stage, four identical cultures from the same sponge were available. After 3 days of culture, the culture medium was completely renewed with fresh culture medium without antibiotic for 24 h prior to stimulation. Old culture medium was spread on Marine growth agar (Difco, Bordeaux, France) to check for bacterial growth. 

In order to study the response of sponge to different LPS, 4-day-old primmorphs were stimulated for 16 h, at 15 °C, with 1 μg mL^−1^ of LPS in a totally renewed cell culture medium [23] either from sponge-isolated bacteria or from *E. coli* (Sigma-Aldrich, St. Quentin Fallavier, France) and without LPS as a control culture. After the stimulation, the primmorphs were directly used for further experiments or stored dried at −80 °C.

### 4.10. Quantitative Reverse Transcription (qRT-PCR) Analysis

Total RNAs were isolated from primmorphs using the extraction solution RNA now (Epicentre, Madison, WI, USA) and DNAse (Epicentre, Madison, MI, USA) according to the manufacturer’s instructions. Five hundred nanograms of total RNAs were used to synthesize first-strand cDNAs using oligodT primers with MultiScribe Reverse Transcriptase (Applied Biosystem, Foster City, CA, USA) according to the manufacturer’s instructions. A negative control was included using total RNA without the MultiScribe Reverse Transcriptase. The mRNAs of interest were quantified by real-time PCR amplification of their cDNAs. The relative mRNA transcript change relative to every LPS treatment was studied for the MPEG-like protein, using the gene Hypoxanthine-guanine phosphoribosyltransferase (HPRT) as a housekeeping gene [7]. PCR reactions were performed in triplicate with the 7300 Real Time PCR System apparatus (Applied Biosystems, Foster City, CA, USA). Twenty mL of reactions contained 10 µL SYBR Green PCR Master Mix (including AmpliTaq Gold DNA Polymerase) (Applied Biosystems, Foster City, CA, USA), 2 µL of each primer (3 mM) and 8 µL of cDNAs. For each experiment, three serial cDNA dilutions were made: 250.0, 25.0 and 2.5 pg mL^−1^. The PCR conditions were 95 °C for 10 min for polymerase activation, and 40 cycles at 95 °C and 60 °C for 60 s and 30 s, respectively. The relative quantification of the mRNAs of interest was obtained by the comparative Pfaffl method using HPRT as endogenous control [7,77]. An ANOVA test was performed as a statistical test with the following hypothesis: the results are different between the controls and the samples or between two samples (*p* < 0.05) using the software Statgraphics^®^ Centurion VII. 

## 5. Conclusions

The effect of LPS from different Gram-negative bacteria was studied on the sponge *S. domuncula* immune system concerning the gene expression of an effective protein, the perforin-like molecule. The LPS from *E. coli* stimulated the expression of the MPEG gene, whereas two Gram-negative sponge-associated bacteria had no effect. The structure of the *Endozoicomonas* sp. HEX311 and *Pseudoalteromonas* sp. 1A1 LPS could partially explain the lack of stimulation. Furthermore, the expression of this gene may be dependent on a complex regulation resulting from the activation of different pathways. The interaction with the LPS interacting protein SLIP from *S. domuncula* seems to rely on the structure of the LPS. Indeed, LPS from *E. coli* did stimulate the expression of the MPEG gene, which is under the control of the SLIP pathways [21] whereas those from *Endozoicomonas* sp. HEX311 and *Pseudoalteromonas* sp. 1A1 did not. The relatively low acylation of the lipid A of *Pseudoalteromonas* sp. 1A1 and *Endozoicomonas* sp. HEX311 was also a possible reason to explain the similar response to both LPS, regarding the regulation of the gene MPEG. They may not activate the LPS receptor SLIP or activate other membrane receptors, also responsible for the regulation of the MPEG gene. Even though there are important differences between the structure and composition of the cell wall components of sponge-associated bacteria, the results of this work reveal that it is not via LPS that the sponge determines the difference between an opportunistic and a commensal bacteria. More experiments could be carried out to further test if LPS do play a role at all by testing more immune response genes, or testing another PAMP.

It is particularly interesting to note that the LPS of the opportunistic bacterium 1A1 did not stimulate the expression of the MPEG gene, whereas this bacterium is able to overpass the host immune system and to become pathogen in some conditions. This strain may have evolved in a way that its LPS mimics the action of that of another species, *i.e.*, commensally sponge-associated bacteria, in order to escape the immune defenses of the host and to remain associated to the sponge. In contrast, when the whole live bacterium 1A1 was experimentally inoculated in the culture medium, it triggered a sponge pathogenic event. It could become pathogenic when the immune defenses of the host are weak and/or when its population is abundant, for example in a confined environment such as an aquarium. 

Further investigations about the sponge immunoreactivity should include several bacteria, bacterial membrane components and molecules from their secretome, alone or in combination with other molecules. Finally, the study of a more consequent number of sponge genes related to the immune system will provide more information to understand the complex interactions existing between associated bacteria and the sponge *S. domuncula* and precisely how it tells the difference between bacteria with different fates.

## Supplementary Files

Supplementary File 1
