# Supplementary material for: Lipopolysaccharides from Commensal and Opportunistic Bacteria: Characterization and Response of the Immune System of the Host Sponge Suberites domuncula
Source: Mar Drugs. 2015 Aug 7;13(8):4985–5006. doi: 10.3390/md13084985 (PMC4557011; doi:10.3390/md13084985)
Supplement: Supplementary File 1 [file marinedrugs-13-04985-s001.docx]

**Supplementary Information**


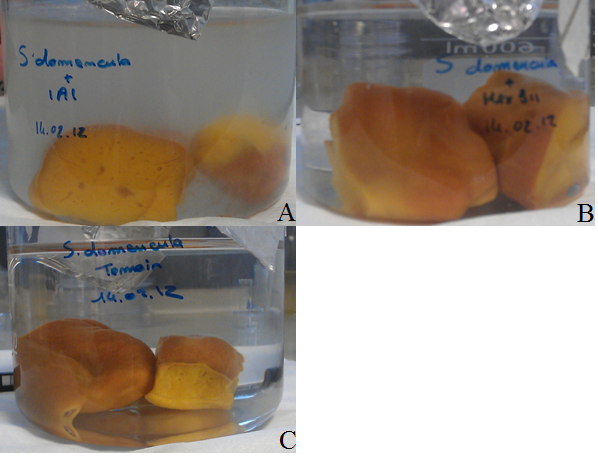


**Figure S1.** Experimental contaminations of the sponge *Suberites domuncula* by the opportunistic bacterium *Pseudoalteromonas* sp. 1A1 (**A**) and the commensal bacterium *Endozoicomonas* sp. HEX311 (**B**). Note the milky aspect of the medium in presence of *Pseudoalteromonas* sp. 1A1 while it remained clear in the presence of *Endozoicomonas* sp. HEX311 as in the control batch (**C**). The results of the experience is presented after 16 h of incubation without agitation, at 15 °C.

© 2015 by the authors; licensee MDPI, Basel, Switzerland. This article is an open access article distributed under the terms and conditions of the Creative Commons Attribution license (http://creativecommons.org/licenses/by/4.0/).
